# Supplementary material for: Automatic beam optimization method for scanning electron microscopy based on electron beam Kernel estimation
Source: Commun Eng. 2024 Jun 20;3:82. doi: 10.1038/s44172-024-00230-3 (PMC11190221; doi:10.1038/s44172-024-00230-3)
Supplement: Supplementary file 2 — Supplementary Information [file 44172_2024_230_MOESM2_ESM.pdf]

Supplementary Information for “Automatic Beam Optimization Method for Scanning Electron Microscopy based on Electron Beam Kernel Estimation”

Yunje Cho[1], Junghee Cho[1], Jonghyeok Park[1], Jeonghyun Wang[1], Seunggyo Jeong[1], Jubok Lee[1], Yun Hwang[1], Jiwoong Kim[1], Jeongwoo Yu[1], Heesu Jeong[1], Hyenok Park[1], Subong Shon[1], Taeyong Jo[1], Myungjun Lee[1], and Kwangrak Kim[1,*]

[1] Metrology and Inspection Equipment R&D Team, Mechatronics Research, Samsung Electronics Co., Ltd., 1-1 Samsungjeonja-ro, hwaseong-si, Gyeonggi-do 18848, Republic of Korea

*Corresponding author: Kwangrak Kim; E-mail: kwangrak.kim@samsung.com; Tel.: +82-10-5154-4805.

Supplementary Algorithm I | Beam Kernel Estimation Process

for i in range (number of sweep images):

$f_i = standard\_normalization\ (f_i)$

$u = standard\_normalization\ (u)$

for n in range(number of iterations):

update u:

$$\frac{\partial F_u}{\partial u} = \frac{1}{n}\sum_{i=0}^{n}\left[\overline{k_{\Sigma_i}} \otimes \left(k_{\Sigma_i} \odot u(x+v_i) - f_i\right)\right] - \lambda \nabla \cdot \frac{\nabla u}{|\nabla u|}$$

$$u \leftarrow u - \epsilon_u \frac{\partial F_u}{\partial u}$$

update kernels:

for i in range (number of sweep images):

$$\frac{\partial F_{\Sigma_i}}{\partial k_{\Sigma_i}} = \left(k_{\Sigma_i} \odot u(x+v_i) - f_i\right) \otimes \overline{u(x+v_i)}$$

$$\frac{\partial k_{\Sigma_i}}{\partial \Sigma_i} = \frac{1}{K}\int_{\boldsymbol{x}} -\frac{1}{2} k_{\Sigma_i}(\boldsymbol{x})\left({\Sigma_i}^{-1} - {\Sigma_i}^{-1}\boldsymbol{x}\boldsymbol{x}^T{\Sigma_i}^{-1}\right)d\boldsymbol{x}$$

$$\frac{\partial F_{\Sigma_i}}{\partial \Sigma_i} \leftarrow clipping\left(\frac{\partial F_{\Sigma_i}}{\partial k_{\Sigma_i}} \cdot \frac{\partial k_{\Sigma_i}}{\partial \Sigma_i}\right)$$

$$\Sigma_i \leftarrow clipping\left(\Sigma_i - \epsilon_\Sigma \frac{\partial F_{\Sigma_i}}{\partial \Sigma_i}\right)$$

$$k_{\Sigma_i} = get\_kernel\_image\_from\_Gaussian\_covariance(\Sigma_i)$$

update v:

for i in range (number of sweep images):

$$v_{i1} \leftarrow -\frac{\sum_{pixels}\left[\left(k_{\Sigma_i} * u_x\right) \cdot \left(k_{\Sigma_i} * (v_{i2}u_y + u) - f_i\right)\right]}{\left\|k_{\Sigma_i} * u_x\right\|_2^2}$$

$$v_{i2} \leftarrow -\frac{\sum_{pixels}\left[\left(k_{\Sigma_i} * u_y\right) \cdot \left(k_{\Sigma_i} * (v_{i1}u_x + u) - f_i\right)\right]}{\left\|k_{\Sigma_i} * u_y\right\|_2^2}$$

$\epsilon_u \leftarrow \eta_{decay} \cdot \epsilon_u$

$\epsilon_\Sigma \leftarrow \eta_{decay} \cdot \epsilon_\Sigma$

Supplementary Algorithm 1 | Algorithm 1 illustrates the beam kernel estimation process, demonstrating the operations required to single update the parameter set of SEM. Please refer to the manuscript for the meanings of each character and operational symbol. The function named *clipping* refers to the process of multiplying a specific scalar with a matrix, preventing the elements of the matrix from exceeding a certain limit in both covariance and its gradient. This method is employed to clip the matrix, mitigating overshooting and enhancing the convergence speed. The function named *get_kernel_image_from_Gaussian_covariance* extracts an image of a specified size of Gaussian kernel using covariance matrix.
