# Supplementary material for: Automatic beam optimization method for scanning electron microscopy based on electron beam Kernel estimation
Source: Commun Eng. 2024 Jun 20;3:82. doi: 10.1038/s44172-024-00230-3 (PMC11190221; doi:10.1038/s44172-024-00230-3)
Supplement: Supplementary file 3 — Description of Additional Supplementary Files [file 44172_2024_230_MOESM3_ESM.pdf]

Description of Additional Supplementary Files

File name: Supplementary Movie 1

Description: A video recording of the automated control process presented in this paper is shown above. The screen on the left side represents an in-house program, showing update of parameter set, while the screen on the right displays live video of the current sample using the Zeiss SmartSEM program. With each update of SEM parameters, the proposed beam kernel estimation process is executed. As a result of these parameter updates, there is noticeable enhancement in the sharpness of SEMimages. Please play slide show to watch video.
